# Acute Stress Attenuates Cognitive Flexibility in Males Only: An fNIRS Examination

**DOI:** 10.3389/fpsyg.2018.02084

**Published:** 2018-11-01

**Authors:** Vrinda Kalia, Karthik Vishwanath, Katherine Knauft, Bryan Von Der Vellen, Aaron Luebbe, Amber Williams

**Affiliations:** ^1^Department of Psychology, Miami University, Oxford, OH, United States; ^2^Department of Physics, Miami University, Oxford, OH, United States

**Keywords:** acute stress, cognitive flexibility, Wisconsin Card Sorting Test, fNIRS, gender differences

## Abstract

Cognitive processes that afford us the ability to control thoughts and achieve goal-directed behavior are known as executive functions. Empirical evidence in the past few years has demonstrated that executive functions can be influenced by acute stress. The impact of acute stress on cognitive flexibility, a key aspect of executive functions, has received little attention in the literature. We present the results of two experiments conducted to examine the effect of acute stress on cognitive flexibility. Acute stress was induced using the cold pressor task. Cognitive flexibility was assessed using the Wisconsin Card Sorting Test (WCST). Across both experiments acute stress had an attenuating effect on task switching on the WCST. Our findings also indicate that this effect was moderated by the participant’s gender. In Study 1, we observed that following stress exposure male participants in the stress condition made more perseverative errors than participants in the control group. In Study 2, we examined the bilateral hemodynamics in the prefrontal cortex (PFC) during acute stress induction using functional near infrared spectroscopy (fNIRS). Our analysis indicated that functional oxyHb signals fluctuated with greater amplitude than systemic components for participants in the stress group relative to those in the control group. In addition, oxyHb levels post stress induction were correlated with performance on the WCST for the male participants in the stress group only. Concordant with previous reports, our findings indicate that acute stress impacts cognitive flexibility in males and females differentially. Our work also demonstrates the feasibility of using fNIRS as a practical and objective technique for the examination of hemodynamics in the PFC during acute stress.

## Introduction

Executive functions are a set of top-down mental processes that afford us the ability to control our thoughts to achieve a goal (Bunge et al., 2002; Diamond, 2013). In the past few decades, research has shown that executive functions develop slowly over time in tandem with a maturing prefrontal cortex (Diamond, 2013). Neurologically, executive functions are coordinated by activity in the prefrontal cortex (PFC) of the human brain (Best and Miller, 2010; Diamond, 2013). Although the exact mechanisms of the cognitive processes that underlie executive functions are active areas of behavioral and psychological research (Testa et al., 2012), there is broad agreement that executive functions include at least the following three components: inhibition, working memory, and cognitive flexibility (Miyake et al., 2000). Inhibition or inhibitory control involves processes that allow the individual to override a habitual or dominant response (Zelazo, 2006). Working memory or monitoring refers to processes that allow individuals to hold information in their mind and manipulate it (Diamond, 2013). Cognitive flexibility is the aspect of executive functions that is often last to emerge in development (Diamond, 2013). Cognitive flexibility, also referred to as shifting, provides the individual with the ability to flexibly change their behavior based on shifting rules, criteria or tasks (Ionescu, 2012). Thus, cognitive flexibility is sometimes used as an indicator of an individual’s ability to adapt to contextual and situational constraints (Goldfarb et al., 2017).

In recent empirical work, researchers have demonstrated that some of the variability in executive functions could be influenced by environmental demands (Shields et al., 2016a; Roos et al., 2017). The implication of this line of research is that executive functions display state-based characteristics as well as stable trait-like attributes (Roos et al., 2017). One such contextual factor that appears to influence executive functions is acute stress (Shields et al., 2016a). Stress is known to threaten homeostasis and stimulate a series of physiological responses in the human body and brain (Arnsten, 2009). Stress may be of two types, chronic or acute. Chronic stress is long-term stress associated with a continued threat or multiple stressors (e.g., ongoing problems in the workplace; Shields et al., 2016a). Acute stress, on the other hand, is a transient and recent experience with a single stressor (e.g., getting a speeding ticket; Shields et al., 2016a). When the acute stress response is initiated, a series of orchestrated processes involving hormones are engaged, all of which are capable of influencing cognitive functions (Elling et al., 2011).

Prior research has shown that the body and brain respond quite differently when experiencing acute vs. chronic stress (Dhabhar and McEwen, 1997; McEwen, 2004). Acute stress responses prepare the body for action (“fight or flight”). This allows the individual to make rapid assessments about threats, respond appropriately within the threatening context, and/or restore homeostasis when the stressor is no longer present (Hermans et al., 2014). Neuroimaging studies suggest that the prefrontal cortex (PFC) is implicated in modulating an individual’s cognitive and emotional response to stress (Cerqueira et al., 2008; Shansky and Lipps, 2013). Yet, research using functional magnetic resonance imaging (fMRI) has demonstrated that acute stress is associated with increased activation of the salience network in the human brain (Hermans et al., 2014). The salience network includes the amygdala, limbic system, dorsal anterior cingulate, and anterior insula (Hermans et al., 2014). Activation of the salience network is accompanied by decreased activation in the dorsolateral prefrontal cortical (PFC) areas during acute stress (Qin et al., 2009). Overall, the neurological evidence appears to indicate that acute stress enhances activation of the salience network but may have a debilitating effect on the executive control network (Hermans et al., 2014).

Although it would be logical to assume that stress attenuates executive functions, the empirical evidence from behavioral studies with humans is riddled with inconsistencies (Plessow et al., 2011; Shields et al., 2016a). Some research has shown that stress impairs aspects of executive function (Schoofs et al., 2009; Shields et al., 2016b) whereas other studies have shown that stress actually enhances components of executive function (Schoofs et al., 2013; Schwabe et al., 2013). One demonstration of effective cognitive control is the individual’s ability to flexibly change behavior under stressful circumstances in order to achieve a goal (Plessow et al., 2011). Yet, the impact of acute stress on cognitive flexibility has not been extensively studied (Plessow et al., 2011; Shields et al., 2016a).

In a recent study with a large sample size (*N* = 113), Shields et al. (2016b) reported on the negative effect of acute stress on cognitive flexibility. Acute stress was induced using the Trier Social Stress Test (TSST; von Dawans et al., 2011) in half the participants. Cognitive flexibility was assessed using the Berg Card Sorting Test (BCST; Berg, 1948), which is a set shifting task available on the online platform PEBL. Shields et al. (2016b) observed that male participants in the acute stress condition, compared to females and participants in the non-stress condition, made more perseverative errors on the BCST. In essence, the researchers reported that acute stress induction impaired cognitive flexibility in men but not in the women in their sample. It is important to note that Shields et al. (2016b) only examined the effect of acute stress on a single measure of cognitive flexibility, perseverative errors—the tendency to persist in the application of a rule that no longer applies (Zelazo et al., 2004; Zelazo, 2006). But, cognitive flexibility is a complex construct and no one behavior can be used as a prototypical indicator of flexibility (Ionescu, 2012).

Traditionally cognitive flexibility has been assessed in the laboratory, using set shifting tasks (Goldfarb et al., 2017). Set shifting tasks generally require individuals to switch between strategies with only one strategy being relevant at any given point in time (e.g., sorting cards by numbers or sorting cards by shape, but only one rule applies in a given trial). Consequently, these set shifting tasks provide information about three related aspects of cognitive flexibility: (1) the ability to switch behavior flexibly based on task demand, (2) the ability to monitor ongoing task demands, and (3) the ability to ignore distracting information irrelevant to task demand. Set shifting is implicated in the PFC, which is impacted by stress (Liston et al., 2006; Hamilton and Brigman, 2015). Therefore by asking individuals to switch between strategies, researchers can capture the additional cost of switching between task demands under stressful conditions (Plessow et al., 2011).

In a thoughtfully designed study, Goldfarb et al. (2017) used the cold pressor task (Lovallo, 1975) to examine the effect of acute stress on cognitive flexibility (measured using a delayed match-to-sample task). They wanted to observe whether an individual’s ability to switch between task demands was attenuated by stress induction. In order to do so they used a within subjects design (i.e., assessing participants cognitive flexibility pre and post stress induction). They were able to show that changes in flexible behavior were associated with stress-induced changes in cortisol levels. Individuals with larger cortisol responses to the stress experience also demonstrated a greater switching cost. Additionally, they observed that males exhibited reduced accuracy in updating aspects of cognitive flexibility whereas females improved in updating. Overall, the findings of this study indicate that it is relevant to account for individual differences in any examination of the relation between stress and cognitive flexibility (Goldfarb et al., 2017).

The relation between acute stress and cognitive flexibility is influenced by two important moderators (1) method of stress induction and (2) sex of the participants (Shields et al., 2016a; Goldfarb et al., 2017; Roos et al., 2017; Shields and Slavich, 2017). Empirical evidence indicates that not all methods of stress induction in the laboratory produce equivalent effects (Smeets et al., 2012; Goldfarb et al., 2017; Shields et al., 2017). Both the nature of the stressor (i.e., physiological vs. psychological) as well as the duration of the stressor (e.g., 3 min for the CPT vs. 15 min for the TSST) can impact the stress response (Smeets et al., 2012). For instance, a socially evaluative stressor such as the TSST is associated with an increase in ruminative thinking after the task (Zoccola et al., 2008). Rumination is a maladaptive form of thinking that is related to higher number of perseverative errors on set shifting tasks (Owens and Derakshan, 2013; Yang et al., 2017).

In addition, an individual’s stress response appears to differ as a function of sex (Kajantie and Phillips, 2006; Lupien et al., 2009). Largely based on animal data, various studies have shown that cortisol levels differ as a function of stress following stress exposure (Kudielka and Kirschbaum, 2005). Yet, the majority of studies on stress effects on the brain and cognition have only male samples (Lupien et al., 2009). Consequently, perhaps, available empirical evidence of executive processes impacted by stress and differentiated by sex is inconsistent (Schoofs et al., 2013; Shields et al., 2016a). For instance, exposure to stress appears to enhance working memory performance in males in comparison to females (Schoofs et al., 2013). But a different pattern of results is observed for cognitive flexibility. The findings of research, with rodent and human models, indicate that stress attenuates cognitive flexibility more in males than in females (Laredo et al., 2015; Shields et al., 2016b). Laredo et al. (2015) have demonstrated that inhibition of the mu opioid receptor in male rodents is implicated in reduced behavioral flexibility following stress. However, partly due to the paucity of studies that have examined this relation in humans, the underlying cause of the exhibited sex difference in cognitive flexibility following stress induction is not understood (Shields et al., 2016a).

One proposed explanation of the relation between stress and cognitive flexibility is that stress reduces cognitive flexibility by taxing attentional resources (Liston et al., 2009). Since flexible behavior is dependent on the effective use of attentional capacities, depletion of key resources results in inflexible behavior (Plessow et al., 2011). Empirical support for this proposition comes from published work indicating that acute stress impairs working memory (Schoofs et al., 2009; Sänger et al., 2014). An alternative proposal suggests that acute stress shifts resources from top-down control processes to more habitual bottom-up processes (Schwabe et al., 2010; Gagnon and Wagner, 2016). Thus, stress enhances the propensity to persist with a particular behavioral response because it unlocks the contingent link between an action and its associated outcome (Schwabe and Wolf, 2011). Research evidence in support of this claim comes from published work indicating that acute stress encourages habitual persistence in individuals (Schwabe and Wolf, 2011; Vogel et al., 2016).

We present the results of two studies designed to examine the impact of acute stress on cognitive flexibility. Study 1 is a behavioral examination of the impact of acute physiological stress (i.e., CPT) on performance on the Wisconsin Card Sorting Test (WCST; Curtiss and Tuttle, 1993). In Study 2, we assessed hemodynamic response associated with the induction of physiological stress using functional near infrared spectroscopy (fNIRS) to explore whether hemodynamic changes in the PFC were associated with task performance on the WCST. Based on prior research (Shields et al., 2016b; Goldfarb et al., 2017) our first hypothesis was that acute stress induction would attenuate cognitive flexibility. Specifically, we expected that individuals in the stress condition would exhibit a greater switching cost (i.e., more perseverative errors on the WCST) in comparison to the individuals in the non-stress condition. Since previous studies have demonstrated that the debilitating effect of acute stress on cognitive flexibility is larger for males than females (Shields et al., 2016b; Goldfarb et al., 2017), our second hypothesis was that males in the stress condition would exhibit a greater switch cost than females in the stress condition and participants in the non-stress condition. A recent study has demonstrated that fNIRS can detect hemodynamic changes occurring during the CPT (Barati et al., 2017) so our third prediction was that hemodynamic signals would significantly differ between the stress and control group in the PFC region for participants in Study 2. Based on the proposal that activity in the PFC decreases with acute stress induction (Hermans et al., 2014) and that cognitive processes underlying cognitive flexibility require the use of resources in the PFC regions of the human brain (Liston et al., 2006) our final prediction was that hemodynamic changes observed in the PFC, using fNIRS, would be related to performance on the WCST. Given that this is the first report, to the best of our knowledge, to explicitly explore the relation between acute stress and cognitive flexibility using fNIRS, we did not hypothesize on the strength or direction of the association.

## Materials and Methods

### Study 1 Participants

One hundred and thirty-one college-aged individuals (Females = 65; ages 18–25 years) completed the cold pressor task (Lovallo, 1975) and the WCST (Curtiss and Tuttle, 1993) and a questionnaire requesting demographic information. In exchange for their participation in the study, individuals were given partial course credit. Individuals were recruited for the study until we reached the maximum number of participants approved by the institutional review board for this project. Majority (80%) of the participants were White (with 6% identifying as Asian or Asian-American, 3% African-American, 3% Hispanic and the rest preferring not to identify their ethnicity).

### Study 1 Measures

#### Cognitive Flexibility: WCST (Curtiss and Tuttle, 1993)

Participants’ cognitive flexibility was assessed using a computerized version of the WCST (Curtiss and Tuttle, 1993). The WCST requires that participants match stimulus cards with one of the four category cards by matching either the card colors, shapes or numbers. Participants are asked to identify the correct card sorting rule solely through trial and error and do not receive any information to the sorting rule in use, but receive feedback (“Correct” or “Incorrect”) on the screen after each sort (Heaton et al., 1993). Once the participant learns the sorting rule, they are expected to maintain it and ignore other irrelevant information. After participants demonstrate they have learned the sorting rule by successfully sorting six consecutive trials, the sorting rule changes randomly and the participants have to discover the new rule to successfully sort the cards again.

The WCST has previously been used to measure executive functions and cognitive flexibility by measuring participants’ abilities to learn new sorting rules and adapt to a new environment (Hassin et al., 2009). The WCST provides several measures of cognition but factor analytic studies have indicated that performance, on the task, can be explained by two or three factors (Goldman et al., 1996; Greve et al., 1997, 2005). Other than perseverative errors, we focused on measures that were known to load onto separate factors (Goldman et al., 1996; Greve et al., 1997, 2005). These were—the *total correct, perseverative errors, completed categories, and failure to maintain set*. The *total correct* score is the total number of trials that the participant sorted correctly. The *perseverative errors* score indicates that the participant persisted with a previously correct, but currently incorrect sorting rule, despite feedback indicating that they had made an error. The *completed categories* score evaluates how many categories of rules (e.g., sorting by color, shape, or number) the participants were able to learn correctly. The *failure to maintain set* score assesses the number of times the participant reverted to using an old, incorrect rule after having clearly demonstrated that they had successfully learned the current (and accurate) sorting rule. Although all WCST measures serve as indicators of executive function, the one most often used as a measure of cognitive flexibility is perseverative errors (Berg, 1948; Heaton et al., 1993; Hassin et al., 2009; Nyhus and Barceló, 2009).

#### Acute Stress Induction: Cold Pressor Test (CPT; Lovallo, 1975)

**FIGURE 1:**
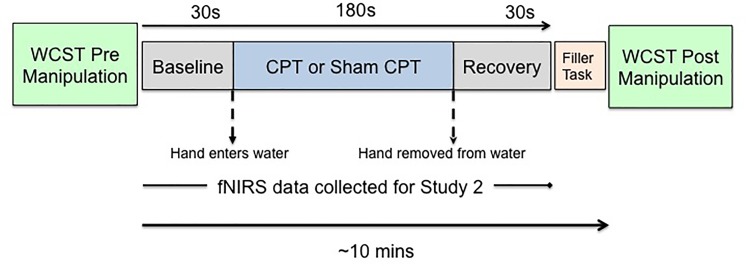
Schematic of the experimental protocol.

Since we planned to measure hemodynamic changes associated with acute stress, we needed to use a method of stress induction that would be compatible with the constraints of data collection using a NIRS (near-infrared spectroscopy) device. As prior research had shown that fNIRS data could be collected effectively with the CPT (Barati et al., 2013). We induced acute stress by administration of the CPT (von Baeyer et al., 2005). The CPT is an experimental technique that has previously successfully been used across multiple studies to induce acute stress (Lighthall et al., 2009; Porcelli and Delgado, 2009; Schoofs et al., 2009). Participants undergoing CPT (Stress group) were asked to placed their non-dominant hand in ice-cold (1–3°C) water for 3 min, while participants undergoing sham-CPT (Control group) were asked to place their non-dominant hand in room-temperature water (∼22°C) water for 3 min. The compliance rate was high, 91% of the participants completed 180 s of the experimental manipulation. Per the protocol approved by the institutional review board, participants were not asked to put their hand back in the water once they had removed it. Participants in both groups were not told for how long the cold presser task would last. An experimenter also observed participants through the task. Both of these experimental choices were expected to increase stress levels since it has been shown that uncertainty, unpredictability, and social evaluation enhance stress responses (Bondi et al., 2008; Schoofs et al., 2009; McGuire et al., 2010). Figure 1 shows a schematic depiction of the experimental protocol.

### Study 1 Procedure

All procedures for the study were completed in the same room. After giving informed consent, participants filled out the demographics questionnaire which provided information about their age, race, sex, and handedness. Per the protocol approved by the institutional review board, participants were checked for obvious injuries and asked about any illnesses or pain sensitivity that may preclude them from participating in the experiment. Once the participants completed the demographic questionnaire, they completed the WCST (Curtiss and Tuttle, 1993) for the first time on a computer. Following the first WCST, participants were randomly assigned to either the Stress or the Control condition. In the Stress condition participants were administered the CPT (Lovallo, 1975) and in the Control condition they were given the sham CPT. After finishing the CPT (or the sham CPT) participants were asked to complete the stress manipulation check and a questionnaire (unrelated to the primary hypotheses). Immediately after this, participants completed the WCST for the second time. Thus, the second WCST was administered approximately 10 min following stress exposure.

### Study 2 Participants

Forty, right-handed, college-aged (18–23 years; *M*_age_ = 19.30) individuals (Females = 20) completed the cold pressor task (Lovallo, 1975), the WCST (Curtiss and Tuttle, 1993) and a questionnaire requesting demographic information. In exchange for their participation in the study, individuals were given course credit. Individuals were recruited for the study until we reached the maximum number of participants approved by the institutional review board for this project. Majority (73%) of the participants were White (10% as Asian or Asian-American, 6% identified as African-American, 6% as Hispanic, and 6% identified as an ethnicity that was not listed.

### Study 2 Measures and Procedure

Materials and measures were the same as Study 1 for the behavioral assessment of cognitive flexibility (i.e., WCST; Curtiss and Tuttle, 1993) and the demographics questionnaire. The compliance rate for the experimental manipulation was high, 100% of the participants completed 180 s of the manipulation. Per the protocol approved by the institutional review board, participants were not asked to complete the manipulation if they removed their hand from the water. All procedures for the study were completed in the same room. Following informed consent, participants filled out a demographics questionnaire, which provided information about their age, sex, and handedness. Consistent with the protocol in Study 1 we checked all participants for obvious injuries and inquired about any illness or pain sensitivity that would preclude their participation. After this participants completed the first manipulation check and the WCST. Next, they were fitted with a fNIRS cap and randomly assigned to either the stress or the non-stress condition. They were administered the CPT or sham CPT, as appropriate. At this point participants completed the self-reported stress levels (or manipulation check) for the second time. Following this, they were given a questionnaire (unrelated to the main hypotheses) and the WCST for the second time. Thus, the WCST was administered approximately 10 min following stress exposure.

**FIGURE 2:**
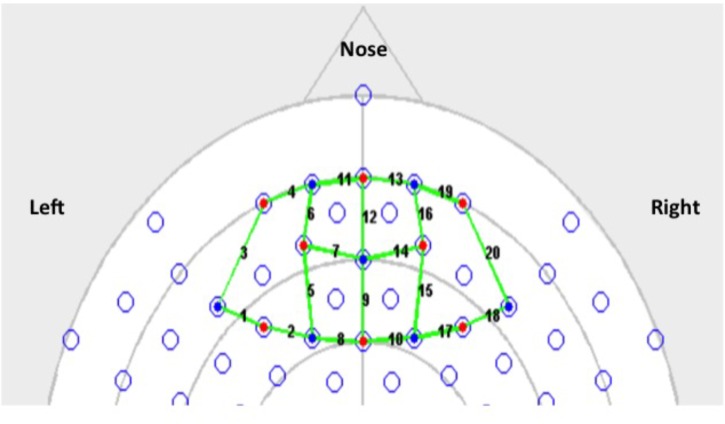
8 by 7 probe layout for the Prefrontal Cortex (PFC).

Since our goal was to examine hemodynamic changes associated with acute stress, fNIRS data were collected for the duration of the CPT or the sham CPT. In order to establish a baseline (Lloyd-Fox et al., 2014), 30 s of fNIRS data were also collected immediately prior to stress induction. Recovery data was collected 30 s immediately post stress induction. The fNIRS data was collected using a 64 channel NIRx Scout system configured using the 8x7 Prefrontal Cortex Layout. Figure 2 shows the topographic probe layout map along with the standard EEG 10/20 coordinate system (open circles). Participants’ heads were individually fitted with a retaining cap (which was selected by measuring the circumference of each participant’s head) and contained all of the source and detector fibers (red: source and blue: detector in Figure 2). This arrangement gave a total of 20 (nearest and second-nearest) measurement channels, with an overall source-detector separation of ∼3 cm (these are marked by lines and numbered in Figure 2; numbers identify each channels).

Each scan acquired by the instrument produced intensity data recoded at each detector location, paired to a specific source location, where each source location had two LED sources centered at 760 and 850 nm. The instrument acquired scans at 7.8 Hz and output power of the sources between <10 mW per wavelength at the surface of the head. Event markers were used to note insertion and withdrawal of participant hands during CPT manually using the NIRStar 12.4 data acquisition system during the process of data collection.

The instrument was calibrated prior to data collection from each participant and Homer2 was used to pre-process the raw intensity signals and convert them into hemodynamic changes as described previously (Huppert et al., 2009; Peng et al., 2017). The experimentally measured intensity counts at the two wavelengths in each channel were converted to relative changes in oxyHb and deoxyHb using the modified Beer–Lambert’s law, and thus yielded relative hemodynamic changes in each channel, across time (Scholkmann et al., 2014). These hemodynamic signals were then corrected to remove motion artifacts (hmrMotionCorrectPCArecurse function) and then bandpass filtered between 0.01 and 0.5 Hz (hmrBandpassFilt function).

As the probe-geometry used to collect these data did not include any short-source separations, we used a previously described statistical method to separate the measured hemodynamic signal into their functional and systemic components (Yamada et al., 2012). As described by Yamada et al. (2012), this method sought to separate the functional and systemic hemodynamic components by minimizing the average mutual information contained between the systemic components of the oxyHb and deoxyHb signals, while asserting a specified negative scalar (*k*_f_) dependence between the functional components of these two variables. We set *k*_f_ = −0.5, for the analysis presented here. This process yielded functional and systemic hemodynamic components in every fNIRS channel, in each participant. The separated components were combined from five channels (for each participant) to localize variations in these hemodynamic signals to four main areas of the PFC. Channels 1–5, 6–10, 11–15, and 16–20 were combined to represent changes in the Left-PFC, Mid-Left-PFC, Mid-Right-PFC, and the Right-PFC regions, respectively (see Figure 2).

## Results and Discussion

### Study 1

#### Manipulation Check: Self-Reported Stress Levels

The self-reported measure assessed stress on a scale of 10 (1 = no stress, 10 = extremely stressed). Participants were administered the stress measure post the manipulation (CPT vs. Sham CPT). An independent samples *t*-test revealed significant differences in the self-reported stress levels post the CPT [*t*(129) = −2.87, *p* = 0.005], Cohen’s *d* = 0.50. Examination of the mean differences for the Stress (*M* = 4.64, *SD* = 2.38) and Control (*M* = 3.52, *SD* = 2.07) groups indicated that the CPT was successful in inducing self-reported acute stress in the participants.

#### Behavioral Results

A 2 × 2 × 2 mixed ANOVA, with Sex (Male vs. Female) and Condition (Stress vs. Control) as between subject factors, and Time of Measurement (Pre vs. Post manipulation) as a within subjects factor, was conducted for each of the four metrics of the WCST. For three of the four WCST metrics, the only significant effect was a main effect of time of measurement. For total correct [*F*(1,127) = 48.89, *p* < 0.001, $\eta_{p}^{2}$= 0.28], completed categories [*F*(1,127) = 10.32, *p* = 0.002, $\eta_{p}^{2}$= 0.08], and failure to maintain set [*F*(1,127) = 8.25, *p* = 0.005, $\eta_{p}^{2}$= 0.06], participants improved in their performance post manipulation, regardless of sex or condition. No significant three-way interactions or any significant two-way interactions emerged. Similarly, there were no significant main effects of sex or condition. Means and SDs are shown in Table 1.

**Table 1:** Means and SDs (in parentheses) of the outcome measures of executive functions on the Wisconsin Card Sorting Test as a function of Condition (Stress vs. Control) for Study 1.

		Total correct	Perseverative errors	Completed categories	Failure to maintain set
**Male Participants**					
Stress Group (*n* = 37)	Before CPT	31.35 (6.63)	4.32 (2.00)	4.14 (1.40)	0.78 (0.89)
	After CPT	34.76 (5.01)	3.54 (1.57)^∗^	4.54 (1.43)	1.14 (1.00)
Control Group (*n* = 29)	Before sham-CPT	30.83 (5.95)	5.28 (2.09)	4.24 (1.53)	0.62 (0.90)
	After sham-CPT	34.70 (5.76)	2.72 (2.15)^∗∗^	5.00 (1.39)	0.69 (0.85)
**Female participants**					
Stress Group (*n* = 20)	Before CPT	30.15 (8.86)	4.55 (2.28)	4.65 (1.50)	0.40 (0.82)
	After CPT	34.90 (6.16)	3.10 (2.13)^∗∗^	4.65 (1.27)	0.95 (1.05)
Control Group (*n* = 45)	Before sham-CPT	30.84 (7.54)	4.44 (2.06)	4.44 (1.53)	0.47 (0.79)
	After sham-CPT	34.09 (5.18)	3.56 (2.05)^∗∗^	4.84 (1.38)	0.73 (1.03)


*^∗^p < 0.01, ^∗∗^p < 0.001; significant result indicates a significant change pre to post manipulation.*

**FIGURE 3:**
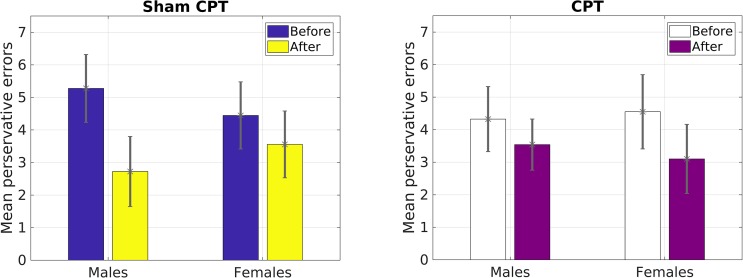
Mean number of perseverative errors as a function of sex and condition in Study 1.

However, for perseverative errors, we observed a significant three-way interaction [*F*(1,127) = 10.37, *p* = 0.002, $\eta_{p}^{2}$= 0.08]. As can be seen in Table 1, there was a significant decrease in perseverative errors made across time for both groups. Based on the report by Shields et al. (2016b), we probed simple effects within sex. For females, there was no significant interaction between condition and time of measurement [*F*(1,63) = 1.29, *p* = 0.26, $\eta_{p}^{2}$= 0.02]. But, there was a significant main effect of time [*F*(1,63) = 22.36, *p* < 0.001, $\eta_{p}^{2}$= 0.26], with females making more perseverative errors before the experimental manipulation than after regardless of condition (see Table 1). For males, there was a significant interaction effect of condition by time of measurement [*F*(1,64) = 11.47, *p* = 0.001, $\eta_{p}^{2}$= 0.15]. Male participants that received acute stress [0.78 fewer errors; *t*(36) = 2.80, *p* = 0.008, *d* = 0.46] did not decrease with the same magnitude as male participants in the control group [2.55 fewer errors; *t*(28) = 5.43, *p* < 0.001, *d* = 1.01]. As Table 1 demonstrates, even though the experimental group made fewer errors prior to the manipulation, this group made more errors than the control group post-manipulation. Thus, male participants in the Stress group exhibited a smaller reduction in their perseverative errors in comparison to the Control group (see Figure 3).

Our primary prediction was supported by the data. The results revealed that acute stress induction attenuated practice effects on the WCST for male participants. We view this finding to be consistent with previous reports demonstrating that acute stress diminishes cognitive flexibility (Liston et al., 2006; Plessow et al., 2012). Although we explored the effect of stress on other measures of general executive function (i.e., total correct, categories completed, failure to maintain set) assessed by the WCST (Greve et al., 2005), we only observed an effect of condition on perseverative errors. Perseverative error is a key indicator of switching costs on the WCST (Nyhus and Barceló, 2009). Thus, results of Study1 provide support for the proposal that acute stress may have a negative impact on task switching but not other aspects of cognitive flexibility (Goldfarb et al., 2017). We believe that the reduced practice effect for perseverative errors demonstrates that male participants in the Stress condition exhibited greater switching costs than female participants in the Stress condition and all participants in the Control condition. This is concordant with the work by Shields et al. (2016b) demonstrating that acute stress impaired cognitive flexibility in males only. We were able to extend the work of Shields et al. (2016b) by inducing stress via the CPT and by using the WCST. Hence, we were able to observe for the first time, to the best of our knowledge, that physiological acute stress increases switching costs in the same manner as a psychological stressor that was used by Shields et al. (2016b).

### Study 2

#### Manipulation Check: Self-Reported Stress Levels

The self-reported measure assessed stress on a scale of 10 (1 = no stress, 10 = extremely stressed). A 2 × 2 mixed ANOVA with Condition (Stress vs. Control) as a between subjects variable and time (Pre vs. Post manipulation) as a within subjects variable was calculated. The analysis revealed a significant interaction effect [*F*(1,38) = 4.96, *p* = 0.03, $\eta_{p}^{2}$= 0.11] but not a significant main effect, *p* > 0.10. Examination of the mean stress scores post manipulation for the Stress (*M* = 3.61, *SD* = 2.03) and Control (*M* = 2.78, *SD* = 1.59) groups indicated that the CPT was successful in inducing acute stress.

#### Behavioral Results: Perseverative Errors

**FIGURE 4:**
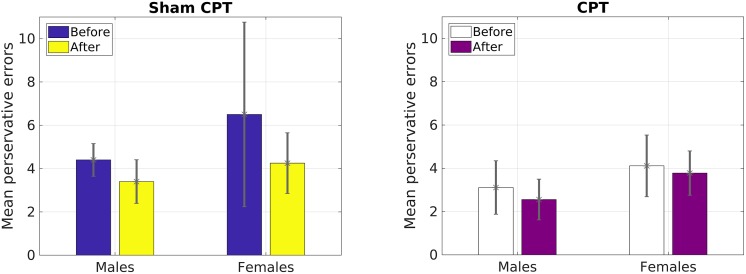
Mean number of perseverative errors as a function of sex and condition for Study 2.

Since the results of Study 1 had indicated that perseverative error was the only variable that was influenced by acute stress, we focused our analysis on that variable for Study 2. A 2 × 2 × 2 mixed ANOVA with Condition (Stress vs. Control) and Gender (Male vs. Female) as a between subjects variable and time of measurement (Pre vs. Post manipulation) as a within subjects variable was calculated. The analysis failed to yield a significant main effect of time [*F*(1,36) = 2.83, *p* = 0.10, $\eta_{p}^{2}$= 0.07]. However, raw values indicated that the decrease in errors on average was larger for the control group (1.86) than the stress group (0.45), which mirrored Study 1. We believe that the non-significance may be due to the reduced power in a smaller sample (see Figure 3). As Figure 4 indicates, male participants in the stress condition generally improved less than participants in the control condition. No other main effects or interactions emerged significant, all *p*s > 0.05.

#### fNIRS Results: Descriptive Analysis

**FIGURE 5:**
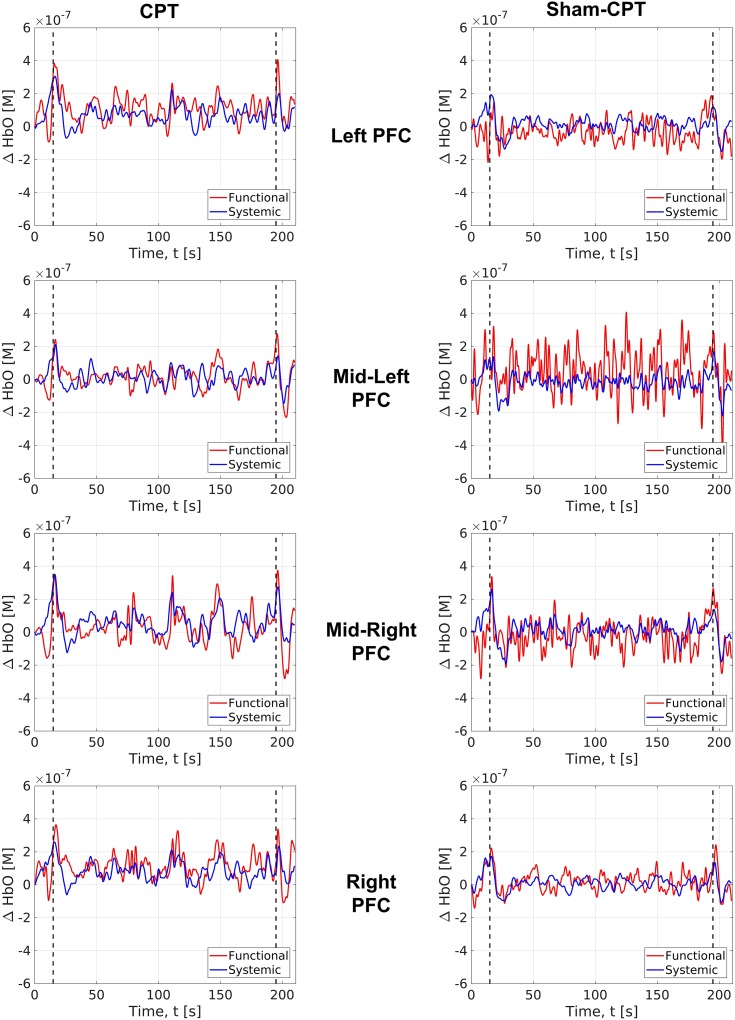
OxyHb levels in the cortical areas (or functional changes) represented by red curves vs. OxyHb levels in the scalp/skull areas (or systemic changes) identified by blue curves. Values represented are averaged across participants in the Stress group (left column) and Control group (right column). The *X*-axis presents the duration of measurement (i.e., time). The *Y*-axis presents the measured levels of OxyHb. 0 in the *Y*-axis represents the start of measurement of OxyHb levels; all changes are assessed relative to this quantity of OxyHb. Top row represents these changes in the Left-PFC region (channels 1–5). Second row represents changes in the Mid-Left-PFC (channels 6–10). Third row represents changes in the Mid-Right-PFC (channels 11–15). Last row represents changes in the Right-PFC (channels 16–20).

As described earlier, changes in raw optical intensity signals (at two wavelengths) from each channel were converted to relative changes in oxyHb and deoxyHb concentrations using the modified Beer–Lambert law (Huppert et al., 2009), each of which were separated to yield functional and systemic components by minimizing the average mutual information between the systemic oxyHb and systemic deOxyHb (Yamada et al., 2012). Functional components of oxyHb indicate changes due to perfusion in the cortical regions, while systemic components represent changes due to blood flow in the scalp/skull. Figure 5 shows the averaged functional (red curves) and systemic (blue curves) components of the oxyHb concentrations, across all participants in the CPT group (left column) and participants in Sham-CPT or Control group (right column). Each row shows these changes obtained from four different spatial locations. Left-PFC region shows data from channels 1–5, the Mid-Left-PFC shows data for channels 6–10, the Mid-Right-PFC shows data from channels 11–15, and the Right-PFC shows data from channels 16–20. For data obtained in the CPT participants (left column), the amplitude of fluctuations in the functional component were larger than corresponding amplitude fluctuations observed for the systemic components. These trends were preserved only in data for the Mid-Left and Mid-Right regions in the Sham-CPT participants. Additionally, the functional and systemic components of oxyHb in the Left-PFC and Right-PFC for participants exposed to Sham-CPT did not exhibit any such large amplitude fluctuations. Given that amplitude fluctuations in the functional component of oxyHb can be interpreted as corresponding changes in cortical perfusion, these data appear to indicate that the CPT stimulus of cold vs. room-temperature water was differentially processed in the Mid-Left and Mid-Right regions of the PFC.

For each participant, the measured time-course for the functional oxyHb change was collapsed into a net change in oxyHb across a specified temporal-window for each of the four PFC regions. This net change in oxyHb was computed in three different time-windows to yield values for the net change in oxyHb at baseline (averaged 10 s before the first stimulus), during the stimulus (averaged during the 180 s of CPT or sham-CPT) and post-stimulus (for 10 s following removal of hand from water) (see Table 2).

**Table 2:** Mean values and SDs (in parentheses) for oxyHb in each of the three windows across all participants, per group (Stress and Control).

PFC side	Baseline	During manipulation	Recovery
**Stress Group (*n* = 18)**			
Left	0.07 (0.19)^∼^	−0.006 (0.02)^∼^	0.06 (0.11)
Mid-Left	0.05 (0.19)	−0.009 (0.02)	0.04 (0.08)
Mid-Right	0.09 (0.14)^∗#^	−0.009 (0.02)^∗#^	0.03 (0.13)
Right	0.08 (0.19)	−0.009 (0.02)	0.07 (0.11)
**Control Group (*n* = 22)**			
Left	−0.009 (0.39)^∼^	−0.005 (0.03)^∼^	−0.03 (0.22)
Mid-Left	−0.07 (1.06)	−0.02 (0.06)	−0.09 (1.05)
Mid-Right	−0.33 (1.39)^#^	0.006 (0.07)^#^	0.20 (1.01)
Right	−0.13 (0.75)	−0.009 (0.04)	0.05 (0.33)


*^∗^p < 0.05. ^∼^Three-way interaction effect between Condition (Stress vs. Control), Gender (Male vs. Female) and levels of oxyHb (Baseline vs. During manipulation), p = 0.12, η_p_^2^ = 0.06. ^#^Two-way interaction between Gender (Male vs. Female) and levels of oxyHb (Baseline vs. During manipulation), p = 0.08, η_p_^2^ = 0.08.*

#### fNIRS Results: Differences as a Function of Stress and Sex

We first sought to determine whether hemodynamic signals for the two groups (Stress vs. Control) differed as a function of Sex (Male vs. Female) and Time of Measurement (Baseline vs. During manipulation). It is relevant to point out that fNIRS measures changes in oxyHb relative to baseline levels. We conducted a series of 2 × 2 × 2 mixed ANOVAs with Condition (Stress vs. Control) and Gender (Male vs. Female) as a between subjects variable and time of measurement (Pre vs. During manipulation) as a within subjects variable. We conducted four ANOVAs to assess changes in oxyHb levels relative to baseline across the four regions of the brain: Left PFC, Right PFC, Mid-Left PFC, and Mid-Right PFC.

A non-significant three-way interaction effect was observed for the Left PFC, [*F*(1,36) = 2.48, *p* = 0.12, $\eta_{p}^{2}$= 0.06] between Condition (Stress vs. Control), Gender (Male vs. Female) and levels of oxyHb (Pre vs. During manipulation). Although not significant at *p* = 0.05, the effect size is similar to behavioral results for Study 1 and Study 2. Non-significant results of paired *t*-tests implied that males and females exhibit dissimilar patterns in relative changes of oxyHb (from baseline to during manipulation) as a function of condition. Males in the Control condition experienced increased levels of oxyHb (*M* = 0.009, *SD* = 0.04) relative to baseline (*M* = −0.09, *SD* = 0.56), *t*(11) = −0.83, *p* = 0.60; whereas females in the Control condition experienced a decrease in levels of oxyHb (*M* = −0.02, *SD* = 0.02), relative to baseline (*M* = 0.06, *SD* = 0.15), *t*(11) = 1.74, *p* = 0.11. However, in the Stress condition males demonstrated a decrease in levels oxyHb (*M* = −0.007, *SD* = 0.02), relative to baseline (*M* = 0.12, *SD* = 0.24), *t*(8) = 1.46, *p* = 0.18. Females also exhibited a reduction in oxyHb levels (*M* = −0.005, *SD* = 0.02), relative to baseline (*M* = 0.02, *SD* = 0.10), *t*(8) = 0.82, *p* = 0.45. Even though the differences we report were not significant at *p* = 0.05, the observed reduction in PFC oxyHb levels during stress exposure in male participants replicates previous fNIRS findings by Al-Shargie et al. (2016; see Supplementary Figures S1, S2). Since, Al-Shargie and colleagues used mental arithmetic (an adaptation of the Montreal Imaging Stress Task; Dedovic et al., 2005) to induce stress in a male-only sample, our work also serves to extend the existing literature.

Second, we observed a marginally significant two-way interaction [*F*(1,36) = 3.15, *p* = 0.08, $\eta_{p}^{2}$= 0.08] between Gender (Male vs. Female) and levels of oxyHb (Pre vs. During manipulation) in the Mid-Right PFC region. Examination of means (see Table 3) indicates that females experienced greater changes in oxyHb, relative to baseline, compared to the males regardless of condition. Paired *t*-tests demonstrated that females exhibited a bigger change in levels of oxyHb during the experimental manipulation (*M* = −0.02, *SD* = 0.02) relative to baseline (*M* = 0.10, *SD* = 0.15), *t*(20) = 3.35, *p* = 0.003. In contrast, the male participants did not exhibit significant changes during the experimental manipulation (*M* = 0.02, *SD* = 0.08) relative to baseline (*M* = −0.41, *SD* = 1.48), *t*(18) = −1.21, *p* = 0.24. Our data may imply that females exhibited a bigger cortical response to the experimental manipulation. There is some evidence to indicate that regulatory control of the hypothalamic pituitary axis (HPA) by the mPFC appears to emerge in the right hemisphere (Cerqueira et al., 2008). So it is possible that the change in oxyHb is a signal associated with increased use of regulatory capacities. However, research has also shown that Right PFC activation is associated with a withdrawal motivation (van Honk and Schutter, 2006). So it could mean that the females in our study were driven to avoid aversive stimuli. More research is needed on the hemodynamic response in the PFC during acute stress before we can arrive at any conclusion.

**Table 3:** Mean values and SDs (in parentheses) for sex differences in hemodynamic activity during and after manipulation per group (Stress or Control) across all participants.

PFC side	Males	Females	Males	Females
**Stress Group (*n* = 18, Female = 9)**				

	**During manipulation**		**After manipulation**	

Left	−0.007 (0.02)	−0.005 (0.02)	0.07 (0.07)	0.05 (0.13)
Mid-Left	−0.005 (0.03)	−0.01 (0.02)	0.09 (0.08)	−0.01 (0.05)
Mid-Right	−0.004 (0.03)	−0.01 (0.02)	0.07 (0.14)	−0.01 (0.11)
Right	−0.004 (0.02)	−0.01 (0.02)	0.04 (0.10)	0.09 (0.11)

**Control Group (*n* = 22, Female = 12)**				

	**During manipulation**		**After manipulation**	

Left	0.009 (0.04)	−0.02 (0.02)	−0.03 (0.31)	−0.03 (0.13)
Mid-Left	−0.02 (0.08)	−0.02 (0.02)	−0.12 (1.59)	−0.07 (0.17)
Mid-Right	0.03 (0.10)	−0.02 (0.01)	0.46 (1.49)	−0.02 (0.11)
Right	−0.007 (0.05)	−0.01 (0.01)	0.07 (0.49)	0.04 (0.12)


Since self-reported stress levels (pre vs. post) had demonstrated that our stress induction was successful, we sought to determine whether hemodynamic signals for the two groups (Stress vs. Control) differed for the duration of the experimental manipulation, relative to baseline. Due to our small sample size we conducted a series of paired *t*-tests, comparing mean oxyHb levels at baseline to average oxyHb during the CPT (or Sham CPT) and to average oxyHb levels post CPT (or Sham CPT) for the two groups separately. As prior research has shown that Left and Right hemispheric regions of the PFC evidence a differential response to stress (Cerqueira et al., 2008), we conducted these analyses for both the Left and Right PFC regions separately. As shown in Table 2 the analyses yielded one significant result, only for the Stress group. Levels of oxyHb in the Mid-Right PFC region differed significantly from baseline levels, *t*(17) = 2.53, *p* = 0.02, *d* = 0.97. Examination of the oxyHb levels for the duration of the stress induction (*M* = −0.009, *SD* = 0.025) indicate a significant drop in oxyHb relative to baseline (*M* = 0.086, *SD* = 0.14). All other comparisons were non-significant, *p* > 0.05. This finding, that acute stress was accompanied by reduced levels of oxyHb in the Right PFC, is consistent with a previous report that used fNIRS to measure hemodynamics of acute psychological stress (Al-Shargie et al., 2016). Overall, the data provide support for the premise that acute stress results in the reallocation of resources away from the executive regions of the brain (Hermans et al., 2014).

#### Correlations Between Hemodynamic Signals and Behavioral Performance

**FIGURE 6:**
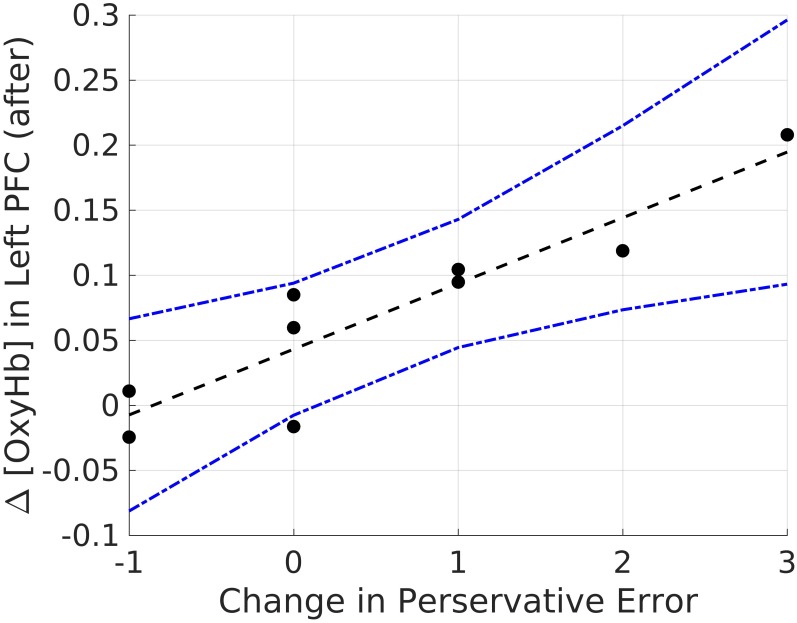
Bivariate correlations between oxyHB and change in perseverative errors for males in the Stress Condition.

Lastly, we explored correlations between hemodynamic signals in the PFC, during the acute stress induction, and cognitive flexibility. In particular, we focused on the change exhibited in task switching (i.e., pre–post perseverative errors) on the WCST. Based on the findings of Study 1, we conducted correlations for male and female participants separately for the two groups (see Tables 4, 5). Since our sample size was small we used a Bonferroni correction, (α_corrected_ = 0.05/8) = 0.00625 to protect from Type 1 error. The analyses revealed one significant correlation between change in perseverative errors (i.e., pre–post) and levels of oxyHb. Specifically, for males in the Stress condition, levels of oxyHb in the Left PFC post stress (i.e., after CPT) were positively correlated with change in perseverative errors (*r* = 0.91, *p* = 0.001) (see Table 4 and Figure 6). In effect, males in the Stress condition who had higher level of oxyHb in the Left PFC following the acute stress induction were also more likely to exhibit a reduction in the number of perseverative errors on the WCST post stress. Thus, higher levels of oxyHb in the Left PFC post-stress correlated with improved performance on the WCST following stress exposure. Our data could indicate that higher levels of oxyHb in the Left PFC after experiencing stress may buffer against the detrimental effects of stress for males. This could mean that recovery from acute stress, for males, may be associated with improvement in cognitive flexibility. This finding is consistent with the proposal that recovery from acute stress may be implicated in the relation between stress and adaptive behavioral outcomes (Geurts and Sonnentag, 2006). However, it’s important to note that we did not observe a relation between oxyHb levels in the PFC and cognitive flexibility in female participants. Additionally, we did not find a significant difference in perseverative errors either as a function of condition (Stress vs. Control) or gender (Males vs. Females).

**Table 4:** Bivariate correlations between oxyHB and change in perseverative errors for male participants as a function of condition (Stress vs. Control), values above the diagonal are for the control group and values below the diagonal are for the stress group.

	Diff. PE	Left PFC	Right PFC	Mid-Left PFC	Mid-Right PFC	Post Stress Left PFC	Post Stress Right PFC	Post Stress Mid-Left PFC	Post Stress Mid-Right PFC
Diff. PE	–	−0.53	0.16	−0.12	−0.33	0.27	0.07	−0.16	−0.27
Left PFC	−0.73	–	−0.49	0.19	0.77	−0.92^∗∗^	−0.40	0.51	0.71
Right PFC	−0.34	0.08	–	0.44	−0.60	0.44	0.95	−0.85^∗^	−0.82^∗^
Mid-Left PFC	−0.18	0.34	0.26	–	−0.43	−0.25	0.25	−0.06	−0.50
Mid-Right PFC	−0.07	0.28	0.39	0.96^∗∗^	–	−0.70	−0.42	0.32	0.87^∗∗^
Post Stress Left PFC	0.91^∗∗^	−0.76	0.31	−0.48	−0.36	–	0.40	−0.45	−0.62
Post Stress Right PFC	0.34	−0.46	0.58	0.04	0.15	0.39	–	−0.81^∗^	−0.62
Post Stress Mid-Left PCF	0.46	−0.59	0.04	−0.59	0.53	0.61	0.52	–	0.69
Post Stress Mid-Right PFC	0.21	−0.44	0.37	−0.53	−0.43	0.45	0.69	0.90^∗∗^	–


*Diff. PE = difference in perseverative errors. Bonferroni correction applied, ^∗^*p* ≤ 0.006, ^∗∗^*p* ≤ 0.001.*

**Table 5:** Bivariate correlations between oxyHB and change in perseverative errors for female participants as a function of condition (Stress vs. Control), values above the diagonal are for the control group and values below the diagonal are for the stress group.

	Diff. PE	Left PFC	Right PFC	Mid-Left PFC	Mid-Right PFC	Post Stress Left PFC	Post Stress Right PFC	Post Stress Mid-Left PFC	Post Stress Mid-Right PFC
Diff. PE	–	0.33	0.36	0.34	0.30	0.18	0.05	0.25	0.09
Left PFC	−0.11	–	0.88^∗∗^	0.81^∗∗^	0.66	0.31	0.15	0.55	−0.00
Right PFC	−0.13	0.36	–	0.95^∗∗^	0.57	0.26	0.13	0.54	0.21
Mid-Left PFC	−0.76	0.27	0.66	–	0.53	0.34	0.29	0.65	0.29
Mid-Right PFC	−0.76	0.19	0.65	0.99^∗∗^	–	0.10	−0.03	0.20	0.24
Post Stress Left PFC	−0.65	−0.23	−0.16	0.42	0.49	–	0.87^∗∗^	0.83^∗∗^	0.59
Post Stress Right PFC	0.07	0.38	−0.57	−0.39	−0.41	−0.02	–	0.81^∗∗^	0.62
Post Stress Mid-Left PCF	−0.54	−0.04	−0.14	0.46	0.44	0.44	0.34	–	0.43
Post Stress Mid-Right PFC	0.21	0.20	−0.06	−0.19	−0.12	0.10	0.47	0.18	–


*Diff. PE = difference in perseverative errors. Bonferroni correction applied, ^∗^p ≤ 0.006, ^∗∗^p ≤ 0.001.*

### General Discussion and Conclusion

An emerging line of research is gathering evidence to prove that individual-level variability in executive functions is influenced by contextual demands. One such contextual force is the experience of acute stress (Roos et al., 2017). Despite the fact that cognitive flexibility is a key component of executive functions (Miyake et al., 2000; Diamond, 2013), and essential to achieving goals (Plessow et al., 2011), few studies have examined the effect of acute stress on cognitive flexibility (Plessow et al., 2011; Shields et al., 2016a). We conducted two experiments to study the impact of acute stress on cognitive flexibility, which was assessed using the WCST. In Study 1, we presented the results of a large sample behavioral experiment wherein we found that acute stress exposure attenuated cognitive flexibility, particularly in the males. Study 2 explored the relation between brain hemodynamics associated with acute stress, measured using fNIRS, and performance on the WCST. Male participants who had higher levels of oxyHb in the Left PFC following stress exposure were also more likely to have fewer perseverative errors post stress. No such association between levels of oxyHb, either during or after stress, and perseverative errors emerged for the female participants. Our data suggest that for males higher levels of oxyHb post stress may buffer against the detrimental effects of acute stress on cognitive flexibility. To the best of our knowledge, this is the first study to report this association between hemodynamic response in the PFC during acute stress induction, assessed using fNIRS, and cognitive flexibility.

Overall, we observed that acute stress has a debilitating effect on cognitive flexibility. Our results are concordant with previous studies that have examined the impact of acute stress on cognitive flexibility (Shields et al., 2016b; Goldfarb et al., 2017). Even though we assessed multiple aspects of executive function, measured by the WCST, the only significant effect of stress emerged with perseverative errors, a key indicator of switching costs (Nyhus and Barceló, 2009). Participants, regardless of condition, improved in their performance on the WCST the second time they completed the task. But participants who were exposed to acute stress exhibited a significantly smaller improvement in perseverative errors. Our work provides additional support for the proposal that not all aspects of cognitive flexibility are negatively impacted by acute stress (Goldfarb et al., 2017). Perseveration is a characteristic example of the failure of executive functions (Zelazo et al., 2004) and can present in many ways (Sandson and Albert, 1984). According to Luria (1965), perseveration, when manifested as the individual’s inability to switch from one task to another is often caused by a dissociation between an action and the intent. Based on this definition of perseveration, our data appear to bolster the proposal that acute stress enhances a persistent behavioral response by breaking the contingent link between action and outcome (Schwabe et al., 2010; Schwabe and Wolf, 2011; Gagnon and Wagner, 2016).

In particular we found that male participants who were exposed to stress exhibited greater switching costs than female participants. This finding is consistent with previous work, examining the effect of acute stress on cognitive flexibility (Shields et al., 2016b; Goldfarb et al., 2017). In addition to behavioral measures, we examined if brain activity during acute stress was related to cognitive flexibility. Our findings indicate that hemodynamic estimates of mean oxyHb levels in the Left PFC post acute stress induction could be related to the change in cognitive flexibility measured before and after exposure to stress. Although the number of males in the Stress condition is small, we found time-averaged oxyHb levels in the Left PFC was positively related to improved performance on the WCST (Table 4). In effect, increased oxygenation in the Left PFC during recovery after acute stress exposure was associated with improved flexibility post stress. This correlation only emerged for the male participants in the stress condition. Our work adds to the growing body of literature demonstrating that stress effects on executive functions are moderated by participant gender (Shields et al., 2016a).

It’s important to point out that in both our experiments we administered the WCST within a few minutes (∼10 min) following stress exposure. This makes it likely that the observed effect was also influenced by adrenergic response evoked by the CPT (Alexander et al., 2007). Often thought of as the first wave of the stress response, the adrenergic response occurs within seconds of exposure to the stressor (McEwen and Sapolsky, 1995) and has a debilitating effect on the PFC (Alexander et al., 2007). Thus, even though our behavioral our data replicate the findings by Shields et al. (2016b), it is possible that the relation between hemodynamic signal in the PFC and cognitive flexibility, that we observed, would change if the WCST were administered after a delay of 20 min post stress exposure.

Consistent with previous research (Barati et al., 2017) our data add credence to the notion that fNIRS can effectively capture hemodynamic responses in the PFC in response to the CPT. We were able to extend the existing literature by nearly doubling the number of participants, adding channels (i.e., covering a substantially larger area of the PFC) and examining correlates with cognitive performance. Although our findings are promising, replication is needed before any firm conclusions can be drawn. Future research should explore whether fNIRS can provide a way to consistently identify an acute stress response.

The findings of our work must be interpreted with the limitations in mind. First, our sample size in Study 2 was moderate, albeit well within the acceptable range for fNIRS studies, and underpowered so caution is warranted when interpreting our data. But, we believe its value was in allowing us to demonstrate that fNIRS is an ecologically valid and effective measurement technique to study the impact of acute stress on PFC activity. Second, we only used self-reported stress levels which are subjective. This limits our ability to make any claims about the intensity of stress experienced by our participants. Future replication of this study with measures of individual differences in stress-related systemic physiological effects (e.g., cortisol, heart-rate variability) is warranted. In addition, we only assessed self-reported stress levels once, post manipulation, for Study 1. Hence, it is possible that individual levels of variability in stress levels prior to exposure might have influenced the observed results. Third, we did not check the participants for smoking behavior, drug intake or caffeine consumption, or phase of the menstrual cycle. Each are established physiological mediators of the stress response (Kajantie and Phillips, 2006; Lovallo et al., 2006). Hence, it is possible that our results were influenced by this lapse. Thus, we cannot draw any firm conclusions from our data until more work is done on this topic. It is also possible that the mean hemodynamic measures used here could be impacted by anticipatory or compensatory physiological activation of neural responses due to the CPT. However, we would expect such compensatory or anticipatory mechanisms to be most dominant only immediately following the stimulus rather than well past the initial exposure. Finally, we only used undergraduate students in our experiments. Since past research has shown that executive functions change with age (Zelazo et al., 2004) our findings may not generalize to older or younger populations. Future research should examine the impact of acute stress on cognitive flexibility with children and older adults. Nevertheless, our work adds to the limited research on the effect of acute stress on cognitive flexibility and by demonstrating the efficacy of fNIRS, in assessing acute stress, provides an additional method of unpacking the mechanistic relation between acute stress and cognitive control.

## Ethics Statement

This study was carried out in accordance with the recommendations of “Institutional Review Board” with written informed consent from all subjects. All subjects gave written informed consent in accordance with the Declaration of Helsinki. The protocol was approved by the Departmental Review Board in the Psychology Department at Miami University, OH.

## Author Contributions

VK developed the study concept and design. BV and KK collected all the data for Study 2. KV, VK, AW, and AL conducted data analyses and interpretation. VK and KV drafted the manuscript, and KK, BV, AL, and AW provided essential additions, revisions, and feedback. All authors approved this version of the manuscript for submission.

## Conflict of Interest Statement

The authors declare that the research was conducted in the absence of any commercial or financial relationships that could be construed as a potential conflict of interest.

## Supplementary Material

FIGURE S1 OxyHb levels in the cortical areas for males. Functional changes represented by red curves vs. OxyHb levels in the scalp/skull areas (or systemic changes) identified by blue curves. Values represented are averaged across participants in the Stress group (left column) and Control group (right column). The *X*-axis presents the duration of measurement (i.e. time). The *Y*-axis presents the measured levels of OxyHb. 0 in the *Y*-axis represents the start of measurement of OxyHb levels; all changes are assessed relative to this quantity of OxyHb. Top row represents these changes in the Left-PFC region (channels 1–5). Second row represents changes in the Mid-Left-PFC (channels 6–10). Third row represents changes in the Mid-Right-PFC (channels 11–15). Last row represents changes in the Right-PFC (channels 16–20).

FIGURE S2 OxyHb levels in the cortical areas for females. Functional changes represented by red curves vs. OxyHb levels in the scalp/skull areas (or systemic changes) identified by blue curves. Values represented are averaged across participants in the Stress group (left column) and Control group (right column). The *X*-axis presents the duration of measurement (i.e. time). The *Y*-axis presents the measured levels of OxyHb. 0 in the *Y*-axis represents the start of measurement of OxyHb levels; all changes are assessed relative to this quantity of OxyHb. Top row represents these changes in the Left-PFC region (channels 1–5). Second row represents changes in the Mid-Left-PFC (channels 6–10). Third row represents changes in the Mid-Right-PFC (channels 11–15). Last row represents changes in the Right-PFC (channels 16–20).
